# Stammering

**Published:** 1841-09

**Authors:** 


### STAMMERING.

Much attention has been directed to this subject, in Europe, during the last few months; and we are glad to see, by a slip from a newspaper, which was handed us a few days since, that Dr. Toland of Columbia, S. C., has recently operated successfully. "The patient," says the editor, "is a boy about seven or eight years of age; and when we saw and talked with him, on Monday last, only three days after the operation, he was completely cured—every vestige of the impediment to his speech being entirely removed." The operation was "simple, and very little soreness was felt three days after;" but the account does not state, and we have not heard through any other source, what one of the various operations recommended was employed. Dr. T. graduated in the Transylvania University about twelve years ago, and is a man of high respectability and promise in the profession.

